# Linking norms, ratings, and relations of words and concepts across multiple language varieties

**DOI:** 10.3758/s13428-021-01650-1

**Published:** 2021-08-06

**Authors:** Annika Tjuka, Robert Forkel, Johann-Mattis List

**Affiliations:** 1 Max Planck Institute for the Science of Human History, Kahlaische Str. 10, 07745 Jena, Germany; 2 Max Planck Institute for Evolutionary Anthropology, Deutscher Platz 6, 04103 Leipzig, Germany

**Keywords:** Word and concept properties, Interdisciplinary database, Cross-linguistic comparison, Test-driven data curation, Psycholinguistic norms, Ratings, Linguistic data

## Abstract

Psychologists and linguists collect various data on word and concept properties. In psychology, scholars have accumulated norms and ratings for a large number of words in languages with many speakers. In linguistics, scholars have accumulated cross-linguistic information about the relations between words and concepts. Until now, however, there have been no efforts to combine information from the two fields, which would allow comparison of psychological and linguistic properties across different languages. The Database of Cross-Linguistic Norms, Ratings, and Relations for Words and Concepts (NoRaRe) is the first attempt to close this gap. Building on a reference catalog that offers standardization of concepts used in historical and typological language comparison, it integrates data from psychology and linguistics, collected from 98 data sets, covering 65 unique properties for 40 languages. The database is curated with the help of manual, automated, semi-automated workflows and uses a software API to control and access the data. The database is accessible via a web application, the software API, or using scripting languages. In this study, we present how the database is structured, how it can be extended, and how we control the quality of the data curation process. To illustrate its application, we present three case studies that test the validity of our approach, the accuracy of our workflows, and the integrative potential of the database. Due to regular version updates, the NoRaRe database has the potential to advance research in psychology and linguistics by offering researchers an integrated perspective on both fields.

**Supplementary Information:**

The online version contains supplementary material available at 10.3758/s13428-021-01650-1.

## Introduction

Psychologists and linguists collect an increasing amount of data for a growing number of languages to describe various properties of words and concepts. However, no resource exists yet where one could compare different properties of words across languages. Given the increased interest in cross-linguistic (multilingual) studies in the field of psychology (e.g., Gibson et al., 2017; Majid et al., 2018; Jackson et al., 2019; Jackson et al., forthcoming), it would be desirable to have a database that unites norms, ratings, and relations of words and concepts that are available for a variety of languages. Recent approaches offer bibliographies that list information on norm databases (Buchanan et al., 2019b; Winter et al., 2017), or unify information on concepts across languages (Speer et al., 2017). In addition, psychologists provide platforms that include norms and ratings on several psycholinguistic criteria with the possibility to create balanced stimulus sets (for English: Wilson (1988), Guasch et al., (2013), and Buchanan et al., (2019a); for German: Heister et al., (2011)). But none of the available resources in psychology include cross-linguistic data on *word and concept properties* from *norm* and *rating* studies. In addition, to our knowledge, no database exists that combines norms and ratings from psychology with data on word relations from comparative linguistics, such as historical linguistics and linguistic typology.

Since linguists study diverse languages from a synchronic as well as a diachronic perspective, linguistic data offers different dimensions of word and concept properties. Data from linguistics include rankings of concepts regarding linguistic constructs such as *stability* (the robustness of the connection between a word and word meaning over time, e.g., Petroni & Serva 2010; Dellert & Buch 2018), *borrowability* (the likelihood that a word is transferred or borrowed from one language to another, e.g., Carling et al., 2019; Vejdemo and Hörberg, 2016), or *polysemy* (the degree to which a word expresses multiple concepts, e.g., List et al., 2018; Rzymski et al., 2020). The relation between words and concepts are usually derived from the comparison of multiple languages, whereas psychological norms and ratings are collected for one particular language. The integration of data from comparative linguistics would allow psychologists to strengthen the cross-linguistic perspective of their discipline (see also Jackson et al., forthcoming).

At the same time, linguists would benefit from having access to norms and ratings collected in large studies from psychology. Calude and Pagel (2011) showed that word frequency counts can account for rates of change in that some words are evolving more slowly than others across the world’s languages. In addition, ratings for valence and arousal facilitate the prediction of the differences in the patterns of emotion words across language families (Jackson et al., 2019). These examples show that both disciplines—linguistics and psychology—have knowledge at their disposal whose combination could answer interdisciplinary research questions. In particular, the comparison of word properties across languages has a big potential for understanding language use.

A recent attempt to compare concepts across languages was enabled by the establishment of the Concepticon project (List et al., 2016).^1^ The Concepticon links elicitation glosses in more than 300 concept lists to more than 3000 *Concepticon concept sets*. A subset of the data currently available in Concepticon is given in Table 1. Each Concepticon concept set consists of a unique identifier (a sequential number), a label (for convenience in English), a definition, a semantic field, and an ontological category. The primary intention of the Concepticon project is to provide stable identifiers for concepts used in the linguistic literature in order to ease the aggregation of data sets from different sources. The link between a Concepticon concept set and an elicitation gloss in a concept list facilitates merging data fast and accurately (List et al., 2018). The Concepticon is thus a collection of lexical *comparative concepts* (Haspelmath, 2010) and builds on the premise—shared by many linguists—that an onomasiological comparison of languages can be done in a straightforward manner. The first Concepticon version (List et al., 2016) already contained data sets that have been compiled for applications in psychology as well as data sets offering *metadata*, such as frequency norms (Brysbaert et al., 2014) and links to WordNet (Fellbaum, 1998; Princeton University, 2010). The Concepticon has already been applied in a large-scale study on word meanings across cultures (Thompson et al., 2020). Since it is a multilingual resource and provides information on semantic fields, the Concepticon can be used to study cultural differences in the structure of certain categories across languages.

**Table 1:** Subset of the Concepticon database. The table gives information on the language for which the data was collected, the data type (tags), and the item number. The Concepticon currently includes 353 concept lists (Version 2.4.0., List et al., 2020a)

Concept list	Language(s)	Tags	Items
Bodt and List (2019)	Kho-Bwa (Tibeto-Burman)	questionnaire, proto-language	664
Walworth and Shimelman (2018)	Vanuatu	basic, areal	215
Hale (1973)	Nepal	questionnaire, areal	1798
Bowern (2012)	Tasmanian	questionnaire	644
Swadesh (1955)	Global	basic	100
Chacon (2014)	Tukano	areal	142
Key and Comrie (2016)	Global	questionnaire	1310
Dunn et al., (2017)	Germanic	historical	12

In comparison to Linked Data resources, the Concepticon is a lexicon for concepts in that it defines language-independent concepts and links them to elicitation glosses which are the basis for questionnaires used in language documentation and comparison. The aim is to establish standardization for concepts and provide a meta-resource for comparative concepts rather than listing vocabulary in an attested language. Thus, the Concepticon differs from resources like WordNet (Fellbaum, 1998; Princeton University, 2010) which can be seen as a dictionary including paradigmatic relations for a single language. WordNet is a well-established resource which has found multiple applications for testing general laws of language (e.g., the Zipf’s meaning-frequency law, see Bond et al., 2019; Hernández-Fernández et al., 2016), creating extended resources (Lehmann et al., 2015; Bond and Foster, 2013), and investigating semantic relatedness (Bao et al., 2021; Boyd-Graber et al., 2006; Budanitsky & Hirst, 2006). Although WordNet-based approaches are effectively computing cross-lingual similarity (Agirre et al., 2009), they work with mere translation equivalents instead of relying on expert judgments on a concept in a given language. The synsets in WordNet reflect the concrete meaning of words, whereas the concept sets in Concepticon indicate the intended denotation range of a given elicitation gloss.

The units of comparison in comparative linguistics and psychology are different. The main construct in comparative linguistics is the concept, which can be translated into words in different languages, whereas psychological norm data collections often contain ratings and similar metadata for individual words of a particular language. The conceptual differences between *words* and *concepts* can be reconciled if one keeps in mind that words tend to have a primary expression that is deeply embedded, especially in experiments asking about specific properties. For example, in the specific question about “wave”: does one think of a wave in the ocean or the wave of a pandemic? In the Concepticon, we are mapping *elicitation glosses* as opposed to *word forms* because the underlying concept lists collected for language documentation and comparison consider them approximations of a concept in a given language. The distinction between ‘word’ and ‘concept’ is often blurred which is illustrated by studies that use word frequencies as a proxy for concept frequencies (e.g., Calude & Pagel, 2011). In psychology, the terms ‘word’ and ‘concept’ are frequently used interchangeably (for a discussion of the use of both terms in linguistics versus psychology, see Murphy, 2002; Jackendoff, 1989; Carston, 2012), and written or spoken words are typically used as stimuli in cognitive science to understand conceptual processing (e.g., Mahon & Hickok, 2016). It is thus implicitly assumed that words are equivalent to concepts, so we infer that linking word lists to the Concepticon is legitimate. A glossary of the different terms and their use in this article is given in Table 2.

**Table 2:** Glossary: Terms occurring in this article and their definitions

Term	Definition
*word*	The term ‘word’ refers to a meaningful unit in a particular language. We are aware of the difficulties in defining the term ‘word’ (Haspelmath, 2011), so we do not aim for an all-encompassing definition. In the present study, words are defined as items in a list that have been evaluated according to a particular property.
*concept*	‘Concept’ is defined as a non-linguistic psychological representation of an object in the world. It includes the knowledge of existing entities and their properties (Murphy, 2002).
*word form*	The term ‘word form’ is the form side of a word understood as a linguistic sign, as given in the orthography or as a sound sequence.
*elicitation gloss*	An ‘elicitation gloss’ is used in linguistic fieldwork to denote a given concept in a language. They are established by linguists and are often based on already existing concept lists. Depending on the source language used for the elicitation, the gloss can be in English, Chinese, Spanish, or any other language.
*Concepticon concept set*	A ‘Concepticon concept set’ (also simply ‘concept set’) consists of a unique identifier, a label, a definition, a semantic field, and an ontological category. The concept identifier (e.g., “1”) is connected to a unique label (e.g., “contemptible”). The following example gives a complete Concepticon concept set: 1 contemptible “Deserving of contempt or scorn.”, Emotions and values, Property. The Concepticon concept sets reflect concepts that are deemed interesting for comparison by linguists and occur frequently in concept lists (List et al., 2016).
*concept list*	The term ‘concept list’ refers to a compilation of concepts in the form of elicitation glosses. They are used by linguists who want to elicit a concept in a particular language. In contrast to dictionaries, the lists are based on questionnaires and are compiled for language comparison or documentation (List, 2018). The list is usually stored in a tabular format in which the elicitation glosses are listed in one column and each row correspond to a concept. Sometimes additional information such as example sentences or ranks are provided.
*word list*	A ‘word list’ is defined as a large collection of items that are evaluated for a particular property. The authors of these lists usually do not provide additional information on the intended meaning of a particular word. The list is stored in a tabular format in which a column corresponds to a property and a row represents an observation for a given word.
*data set*	The term ‘data set’ is used when referring to a word list in addition to its metadata, i.e. the list, the scripts for mapping the list, and the raw data.
*word and concept properties*	‘Word and concept properties’ are variables that are collected in psychology and linguistics including psycholinguistic measures, network relations, among others.

The Database of Cross-Linguistic Norms, Ratings, and Relations for Words and Concepts (NoRaRe) builds on the Concepticon to provide convenient access to data from linguistics and psychology across a variety of languages. By using Concepticon as a starting point, NoRaRe currently contains 98 data sets with additional information on word and concept properties across 40 languages. Furthermore, the database facilitates a cross-linguistic comparison of many properties. The NoRaRe database can be conveniently extended due to computer-assisted data curation workflows and it is released in regular version updates. The collection is accessible through a software API (written in Python) that allows to test the data for internal consistency and at the same time, offers quick access to the data. Furthermore, we provide a web application so that other researchers can easily examine the data.

Linking word and concept lists with different norms, ratings, and relations across multiple languages is a challenge. In the next section, we elaborate on the data we found and why one cannot compare them directly. To solve the challenges, we established computer-assisted data curation workflows which are des- cribed in Section “Data curation and technical approach”. The scope and concrete use cases of the NoRaRe database illustrate the potential of our approach (Section “Validation”). Finally, we discuss the application and future plans for the new database in Section “Discussion and Conclusion”.

## NoRaRe data overview

Data for word and concept properties are remarkably abundant and diverse. To get a better grasp of the different types of data in NoRaRe, we have divided the data into three distinct groups: *norms*, *ratings*, and *relations*. While norms and ratings are predominantly collected in psychology, most of the data that contain relations come from linguistics. But not only the content of the data varies, they are also stored in different formats and are to a greater or lesser extent accessible.

### Data types: Norms, ratings, and relations

The data type *norms* includes data that are determined by taking samples from a total quantity, for example, counts of word occurrences in a corpus (i.e., word frequency). They are collected and applied predominantly in the field of psychology.^2^ The norms we encountered in the literature include data on word frequencies in subtitles for several languages, for instance, English (Brysbaert & New, 2009), Spanish (Cuetos et al., 2011), Chinese (Cai & Brysbaert, 2010), and Dutch (Keuleers et al., 2010). Additionally, we classified reaction time studies (e.g., Tsang et al., 2018; Ferrand et al., 2010) as norms. Most of the lists of this data type are based on a broad text or word basis and are rarely compiled for smaller languages due to the lack of available sources (an exception is Calude & Pagel, 2011).

*Ratings* are based on participant judgments of a given word in a particular language either on a scale or on other measures, for instance, the age at which a word was acquired. Numerous studies collected ratings, for instance, on age-of-acquisition (e.g., Alonso et al., 2015; Kuperman et al., 2012; González-Nosti et al., 2014). Other studies include ratings for valence and arousal (e.g., Stadthagen-González et al., 2017; Warriner et al., 2013; Yao et al., 2017), perceptual and motor modality (e.g., Lynott & Connell, 2013; Lynott et al., 2020; Díez-Álamo et al., 2018), or discrete emotions (e.g., Briesemeister et al., 2011; Ferré et al., 2017; Hinojosa et al., 2016). Most studies are conducted with speakers of well-documented languages, such as English, Dutch, or Spanish, which is typical for psychological research. The over-representation of data from a Western, educated, industrialized, rich, and democratic (weird) population is striking (Henrich et al., 2010; Jones, 2010). In recent years, linguistic diversity has been increasing, as shown by the publication of ratings on arousal, valence, and discrete emotion for Turkish (Kapucu et al., 2018) or age-of-acquisition ratings for a diverse set of languages from Afrikaans to Western Armenian (Łuniewska et al., 2016; Łuniewska et al., 2019). However, so far, there is no possibility to compare the same property in multiple languages.

The data type *relations* includes, for example, stability rankings, semantic field categorization, and semantic networks. Data on relations are collected predominantly in the field of comparative linguistics which deals with various questions related to the evolution of languages (historical linguistics) and the general properties of the world’s languages (linguistic typology). In addition, data on relations are collected for Natural Language Processing (NLP) and other data-driven fields. Typical examples for relations are lists in which items are *ranked*, *tagged*, or directly *associated* with other items in the same list. In *ranked lists*, words and concepts are ordered by cross-linguistic categories, such as borrowability (e.g., Tadmor, 2009 ) and stability (e.g., Calude & Pagel, 2011). In *tagged lists*, a given word or concept is described by a tag or a set of tags, and different words and concepts can be compared by means of the tags they share (the list of headwords and senses by Starostin (2000) is a classic example). Lists providing concept *associations* are most typically represented by the WordNet ontology (Fellbaum, 1998; Princeton University, 2010). In contrast to WordNet, Vulić et al., (2020) present a large-scale resource with human judgments on semantic similarity for 12 typologically diverse languages that is applied in representation models for NLP tasks. But association data, such as the Edinburgh Associative Thesaurus (Kiss et al., 1973), also fall under this data type as does the recently proposed data sets of cross-linguistic colexifications^3^ (Rzymski et al., 2020). Studies on word and concept relations often only include a small number of items compared to norm and rating studies. However, the items are carefully selected and chosen based on their comparability across multiple languages, including many languages that are notoriously underrepresented in cross-linguistic studies.

### Comparability and availability

According to Wilkinson et al., (2016), data should be *findable*, *accessible*, *interoperable*, and *reusable* (fair). While it is becoming more common to add a section introducing the supplementary material of a given study, some journals obscure the access to the repositories in which data sets are stored. The fact that the data sets are archived on a journal’s website is also problematic. Journals are not properly equipped for long-term archiving, licensing, and regular release updates for the data. The best practice for storing one’s data is, therefore, scientific archiving services, for instance, Zenodo^4^ or the Open Science Framework^5^. These possibilities enjoy increasing popularity (studies that store their data on one of the two archives: e.g., Kapucu et al., 2018; Lynott et al., 2020; Rzymski et al., 2020).

**Fig. 1:**
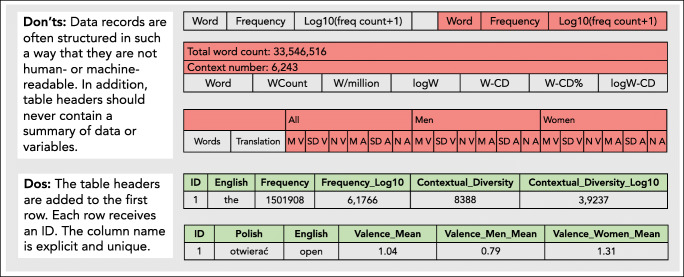
Best practice examples for structuring data sets. The data should be in a machine-readable form. Additionally, the information of the column content should be easily understandable by other researchers (for details on how to structure data in R, see Wickham, 2014)

Even if data can be easily found and accessed, this does not necessarily mean that they can be used and reused. Most data sets presenting word and concept properties are available in the form of tabular data. In a spreadsheet, words or concepts are given in a row of the table, and properties are listed in additional columns. Metadata regarding the content of each column, however, is often lacking. Other researchers who would like to apply the data have to guess the nature of the content based on the table headers. This issue is illustrated in Fig. 1. Since many data sets offer similar norms, ratings, and relations for words and concepts, it would be highly desirable to have *uniform exchange formats*. In addition, a clear licensing policy with open licenses should be provided to ensure that the data can be reused in other studies as well. While many data sets are published without a license, some data sets have a license that explicitly restricts building upon the data or use them in other scientific studies.

Concepticon and NoRaRe are developed as part of the Cross-Linguistic Data Formats Initiative (CLDF, see Forkel et al., 2018), which seeks to standardize various kinds of cross-linguistic data by using ‘CSV on the Web’ (CSVW) and dedicated Python machinery as the basis upon which the standard is built. Both databases are online available, freely accessible, and formatted in CSVW which makes them interoperable and reusable. The advantages of having data on a wide range of word and concept properties stored in this way are that we can compare, evaluate, and answer interrelated questions. Furthermore, studies can be carried out more rapidly and gaps, as well as inconsistencies, become apparent. But the clearest benefit would be the possibility to link data to other resources and make them cross-linguistically comparable.

## Data curation and technical approach

The NoRaRe database is comprised of 98 data sets with 65 word properties across 40 languages (Table 4 provides an overview and the Supplementary Material give a complete list of the data sets). We made the diverse data sets comparable with each other by (1) normalizing the raw data, (2) linking the concepts and words to the Concepticon database, and (3) classifying and labeling the word properties provided by each study. NoRaRe is an extension of the Concepticon that was previously only sporadically linked to metadata on word and concept properties (List et al., 2016). The scope of both resources will continue to grow and it is, therefore, important to establish workflows that allow us to easily curate the available data.

**Fig. 2:**
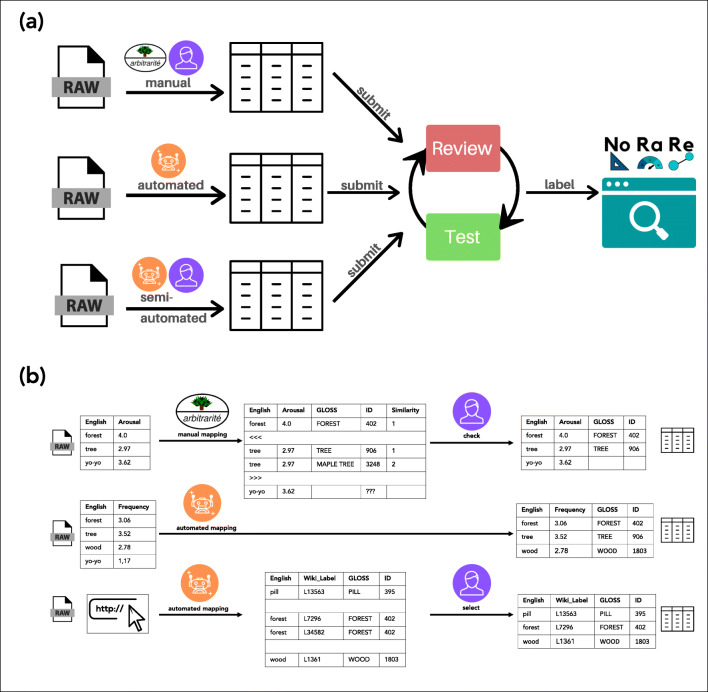
Workflows for data curation. **a** How raw data are converted to unified tabular data formats and consecutively labeled. **b** Details for the individual steps involved in the linking of the different data to Concepticon

The NoRaRe database distinguishes three basic types of word and concept properties: norms, ratings, and relations. The data come from two research fields, namely psychology and linguistics. The data vary considerably in their size from lists with 100 items up to more than 100,000 items. Most data are stored in a discrete form, such as tables, but with little consistency. Another type of data is not available in discrete form, and can only be queried, for example, through a website. These data include word properties from online resources such as Wikidata^6^ or BabelNet (Navigli and Ponzetto, 2012)^7^. Thus, we developed three workflows: (1) a manual workflow for discrete lists up to 2000 items, (2) an automated workflow for discrete lists with more than 2000 items, and (3) a semi-automated workflow for online resources, where we use automatically generated queries that are manually checked (for an overview of the different workflows, see Fig. 2).

All three workflows yield a unified output: either all or a certain part of the items (words or concepts) in the original data are provided in a tabular format along with the information on word and concept properties, and—where available—a link to the corresponding Concepticon concept sets. The tabular format in which we provide the data is strictly standardized, following the recommendations of the W3C for tabular data on the web (Tennison, 2016), also known as CSVW (for details about the use of CSVW for linguistic data, see Forkel et al., 2018). The core idea of CSVW is to increase the interoperability of tabular data by adding metadata in JSON format that conforms to specific recommendations. The CSVW Python package (Bank & Forkel, 2018) allows to automatically test for consistency as well as parse and manipulate data that is conforming to the CSVW recommendations.

**Fig. 3:**
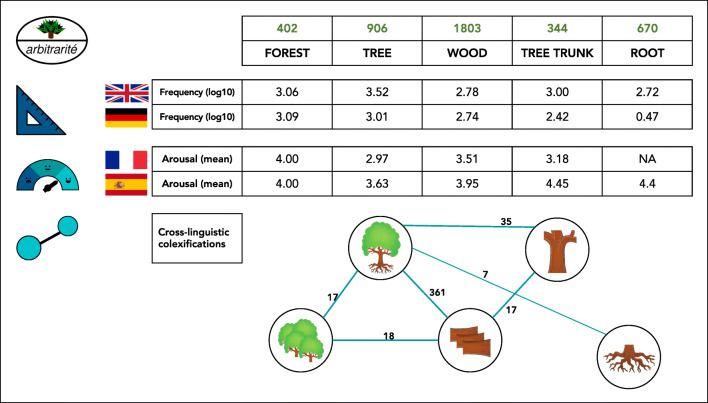
Comparing different kinds of data on word and concept properties as they have been proposed in the literature. **a** The Concepticon on top of the figure offers standardized concept sets for more than 3000 concepts. **b** The SUBTLEX data sets offer frequency counts for words across different languages based on subtitles (Brysbaert & New, 2009; Brysbaert et al., 2011). **c** User-rated collections of psychological categories, such as arousal, have been published for different languages (e.g., Riegel et al., 2015). **d** The CLICS database allows estimating the semantic similarity of concepts by measuring how often they are colexified in the languages of the world (Rzymski et al., 2020)

After the data have been normalized and converted to a tabular format, all data sets are reviewed. To additionally minimize errors, specific tests that check the formal requirements for the data are carried out, based on unit test facilities as they are typically used for the testing of code in software development. Once a data set has passed this *test-driven data curation* process, the word and concept properties provided by a particular data set are *classified* and *labeled* in order to make them comparable against other data sets. Figure 2(a) provides a schematic overview of the data curation. Since we use git for version control and GitHub for data curation, and Zenodo for data storage, all stages of the data curation workflow are transparently documented and can also be directly inspected by anybody interested in the details. Figure 3 provides an example of the resulting cross-linguistic resources that offer data on norms, ratings, and relations across languages for the concept sets of the Concepticon database.

### Workflows

We decided to establish three workflows to account for the different structures that we found for data on norms, ratings, and relations of word and concept properties.

#### Manual workflow

Given that the Concepticon resource already links to all kinds of concept lists of different sizes, purposes, and languages, it is straightforward to use the well-established data curation workflow to link small to moderately large data sets (< 2000 items) providing norms, ratings, and relations. While most of the concept lists released with the first version of the Concepticon (List et al., 2016) were linked by hand, the growing body of elicitation glosses from different languages made it possible to add an automated mapping algorithm in later versions of the Concepticon. The algorithm checks a given elicitation gloss against its hand-curated mapping to Concepticon concept sets. Currently, the algorithm can be carried out in 30 languages and is provided along with the pyconcepticon Python package which also allows testing the data for internal consistency (Forkel et al., 2019). For individual concepts, users can consult a web-based lookup tool that offers a slightly simplified mapping algorithm that currently supports seven languages (List et al., 2018). In addition, users who want to contribute can consult tutorials for different levels of expertise (Tjuka 2020a; Tresoldi 2019a, b).

The Concepticon deals primarily with *concept lists* that need to be distinguished from *word lists*. In a typical concept list, scholars try to assemble different concepts by means of *elicitation glosses* in a certain language in order to express the meaning of the concept they want to list. Since concept elicitation has never been standardized (and the Concepticon aims to provide concept sets with stable definitions and identifiers), it is at times difficult to decide how to interpret the intended meaning of a specific elicitation gloss. This becomes even more difficult when dealing with word lists, where no attempt was undertaken to distinguish between the different meanings of a word. While mapping items in large word lists to Concepticon concept sets, we therefore assume that the most frequent or most prototypical use of a word is intended. For small concept lists, we usually have more information, so we can infer the meaning of an elicitation gloss more precisely (for a discussion on how to decide on a mapping, see Tresoldi 2019a, b). Thus, the description of the Concepticon concept set defines the intended denotation range of an elicitation gloss, for example, the concept set wave (ID: 978) is defined as ‘the concrete wave of water’ rather than ‘the metaphorical wave’. To avoid errors when adding new concept lists to the Concepticon, each new list is accompanied by an extensive review that is conducted independently by the Concepticon editors, a team of linguists (the review process is described in Tjuka, 2021). Since most word lists compiled for studies in psychology do not provide any information on potentially intended meanings of a word, we decided to leave ambiguous cases unmapped instead of mapping them incorrectly to a specific Concepticon concept set. In those cases, a given word does not have a mapping to a Concepticon concept set. Figure 2(b) contrasts the manual workflow with the automated and semi-automated workflow.

The advantage of the manual workflow is that each list is checked carefully by experts and individual mappings can be discussed. To expand the languages in Concepticon, we frequently add concept and word lists in languages other than English. A major achievement was the addition of Multi-SimLex with similarity judgments for 12 languages (Vulić et al., 2020). In the process of mapping the list, we found inconsistencies in the data itself, which is another indication that more rigorous scrutiny of linguistic data is needed (for details on how Multi-SimLex was mapped to Concepticon, see List, 2021).

#### Automated workflow

The detailed manual workflow, including extensive review by the Concepticon editors, is not feasible for data sets with more than 2000 items. In order to make it possible to have access to the specific word properties offered by large data sets, we decided to set up a new algorithm for linking to Concepticon concept sets which is implemented in Python. The basic idea of the mapping algorithm is to employ all previous links available from the Concepticon and order them by priority to check for direct matches against a specific data set. The algorithm consists of three steps. First, all Concepticon mappings for a given language are assembled and ranked according to their frequency of occurrence throughout the concept lists linked to the Concepticon. In a second step, the algorithm iterates over each item in the target data set and checks if the item can be found in the list of assembled mappings. If this is the case, the item will be appended to the list of *potential links* for a given Concepticon concept set. Third, the algorithm iterates over all Concepticon concept sets for which a link was identified and selects one, according to the priority rank.

As an example, consider the English word *wave* which occurs as an elicitation gloss linked to two Concepticon concept sets, namely 918 wave and 3544 wave (verb). While *wave* occurs as an elicitation gloss 19 times for the verb meaning in the Concepticon data, it has been linked 18 times to the noun reading (918) and only once to the verbal reading (3544). The verbal reading, in this case, is justified since the database in which the reading occurs explicitly deals with verbal meanings (Kibrik, 2012). Given that *wave* refers to the Concepticon concept set wave in the overwhelming majority of cases, the algorithm will ignore the verbal reading and link the word to the concept set 918 wave. To further increase the precision of this procedure, it is possible to add part-of-speech information, when available, to give preference in matches for the same part-of-speech.

Although the mapping algorithm can be invoked directly from the command line, we decided that the process needs to be more neatly integrated into a data curation workflow since the results are no longer manually reviewed. For this reason, we established an automated workflow based on Python scripts that can be invoked with the help of a new Python package: pynorare (List & Forkel, 2020). The package also automatizes the download of the data from dedicated URLs and the conversion of individual formats to the standardized tabular format that we employ for all lists. With this workflow, each data set receives a custom Python script that can be called from the pynorare Python library. The script downloads the data set, unpacks it (if needed), pre-processes the data (if needed), and maps the items in the list automatically to the Concepticon concept sets. Figure 2(b) contrasts the automated workflow with the manual and the semi-automated workflow.

By offering users to download the data themselves with the help of our Python library, we contribute to the *reusability* of the data. The data included in the NoRaRe database was either stored in the supplementary material of an article on a journal’s website, in cloud services (OSF or figshare), or on openly accessible websites provided by the creators of the data sets. The automated workflow is simple, fast, and uncomplicated. Once a new large data set is discovered, all that is required is to set up a new Python script that automatically downloads the data set and links it to the Concepticon concept sets using the commands norare download and norare map. The ease of use means that the NoRaRe database can be constantly expanded and lists with 100,000 or more words can be mapped in no time.

#### Semi-automated workflow

There are certain data sets that cannot be easily downloaded and treated with the workflows described in the previous sections. Typical obstacles are their size (sometimes megabytes or even gigabytes), their availability (web-services only), or their structure. While the former two are technical obstacles, the problem of the structure may pose a direct issue. For example, a search for the item *foot* on OmegaWiki^8^, results in three possible senses, namely (1) ‘The part of a human’s body below the ankle [...]’, (2) ‘A unit of measurement equal to twelve inches [...]’, and (3) ‘The lowest support of a structure’. When linking the Concepticon concept set 1301 foot to OmegaWiki by hand, we would select the first over the second and the third option.

It is entirely possible to manually search large databases such as OmegaWiki to find matches to Concepticon concept sets, but we decided it would be easier to develop a semi-automated workflow using software APIs provided by individual online databases. This gives us the possibility to query the data and later manually choose which of the three or more possible matches should be the preferred one. So in the example with the possible meanings of the word *foot* in OmegaWiki, the algorithm would present all three possibilities and the third option would be chosen manually. This procedure differs from the manual workflow used in the Concepticon project. For Concepticon, all data are curated with the manual workflow. However, the manual workflow is not feasible for online databases due to their size. Therefore, we opted for a semi-automated workflow that uses an algorithm based on the hand-curated mappings in Concepticon. The algorithm finds the closest counterparts for our Concepticon concept sets in large semantic databases. A list of possible matches is then created, which is reviewed and the best mapping is selected or, if the mapping is incorrect, deleted. Figure 2(b) contrasts the semi-automated workflow with the manual and automated workflow.

Since online databases do not exist in a tabular format and are often complex, the semi-automated workflow offers the possibility to integrate metadata on words and concepts in the NoRaRe database. This is becoming increasingly important as open resource projects such as Wikidata (Nielsen, 2020) or WordNet (Fellbaum, 1998; Princeton University, 2010) are frequently applied in NLP tasks.

### Web application for accessing NoRaRe

**Fig. 4:**
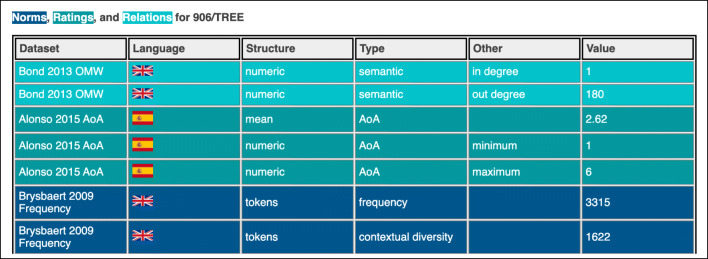
A screenshot of the NoRaRe web application (https://digling.org/norare/) illustrating the values for the Concepticon concept set 906 tree across three different data sets (Bond and Foster, 2013; Alonso et al., 2015; Brysbaert & New, 2009)

Standardizing and linking data sets alone do not guarantee that word and concept properties can be compared across different languages. Additionally, the data need to be labeled and tagged for convenient access and comparison. We established several labels and tags for the different data types, which structure the data into different groups. The result is a categorization of each data point into *language*, *structure*, and *type*. The labels reoccur in the web application of the NoRaRe database.^9^ Figure 4 illustrates a subset of the labels for the concept set 906 tree across three different data sets shown in the NoRaRe web application.

When accessing the NoRaRe web application, one can look up a particular concept in different languages and see in which data sets it occurs. Each data set receives multiple tags, depending on which properties are included. For example, the data in Alonso et al., (2015) is tagged for *AoA* (age-of-acquisition) and the rating results are divided into the *mean*, *minimum*, and *maximum value*. It is also possible to get a general overview of the data sets currently available in NoRaRe via the web application. Under the tab Datasets a list appears and each data set shows up with a label for the *data type* (norms, ratings, or relations) and the *language*.

As of yet, 65 different properties from 98 data sets are available in NoRaRe. In addition to the labels and tags, the data received detailed descriptions for each data point consisting of information about, for example, the scale that was used in a certain rating study, the part-of-speech (i.e., noun, verb, adjective) of the words, the particular subset of the data e.g., male, female, higher education, lower education). The NoRaRe database provides all relevant information so that a convenient comparison across a variety of data sets is possible. The web application is intended to give a clear presentation of the available data and in the GitHub repository, more information about the property in a given data set can be found.^10^

In the future, the NoRaRe database will be integrated into the Cross-Linguistic Linked Data (CLLD) framework^11^ so that the web application is converted into the familiar online appearance similar to the Concepticon web interface.

### Validation

The data curation workflows that we established to add data on norms, ratings, and relations to NoRaRe proved to be very effective. With the pre-defined workflow for linking concept lists to Concepticon (i.e., the manual workflow), we were able to add 15 new data sets^12^ with small numbers of words (< 2000 items) within the first seven months. The new data sets were included in the release of Concepticon Version 2.4.0-rc.1 in July 2020 (List et al., 2020b) and in the release of Concepticon 2.4.0. List et al., (2020a), additionally 22 new lists were included.^13^ For the larger discrete data sets (> 2000 items) and data from online databases, prepared with the automated and semi-automated workflows, we created a new GitHub repository.^14^ The first commit to this repository was on March 31^st^, 2020 and since then we added 43 data sets with the automated and five data sets with the semi-automated workflow. The total number of 48 data sets uploaded within the first four months demonstrates that the data collection can be expanded in a short amount of time (in our case, approx. 10–15 data sets per month). Version 0.1 of the NoRaRe database was released on July 23^rd^, 2020 (Tjuka et al., 2020) and included 71 data sets. The next version (v0.2) was released on March 30^th^, 2021 in which the number of data sets amounted to 98 (Tjuka et al., 2021). As can be seen from this progress, the NoRaRe database will continue to grow in the future.^15^

While steadily adding more data to Concepticon, we are able to expand the scope of the links from Concepticon concept sets to multiple languages. In its current state, the Concepticon includes seven glossing languages (English, German, Chinese, French, Spanish, Russian, Portuguese). Our aim is to broaden the variety of languages and the available mappings across languages in the future. To achieve this goal, we are adding new data sets that continue to expand the number of languages as well as the number of concept mappings across languages (e.g., List, 2020; 2021).

The Python package pynorare was established and developed parallel to the NoRaRe database. It was expanded and adapted to account for the challenges of bringing completely different data set formats into a standardized format. The first release of pynorare Version 0.1.0 was on July 13^th^, 2020. The next version update which included more tests for the data curation was uploaded on July 21^st^, 2020 (List & Forkel, 2020).

The timeline of the releases for Concepticon, the NoRaRe database, and the associated Python package shows that our workflows can be applied, constantly improved, and expanded. The longevity of the Concepticon project ensures regularly updated data. The Concepticon database allows for advancement in cross-linguistic comparison and the development of features such as the NoRaRe collection. Therefore, it adds value to research disciplines like psychology by offering deliberately curated data on word and concept properties.

### Descriptive statistics of NoRaRe

The results of our efforts for test-driven data curation are 65 unique word and concept properties derived from 98 different data sets across 40 languages collected in the current version of NoRaRe. Sixteen out of 98 reflect *norms* in the notion defined above, 54 reflect *ratings*, and 34 belong to our data type *relations* (note that some data sets include multiple data types). Table 4 provides an overview of a small part of the data and also shows how many words and concepts we managed to link to our Concepticon concept sets.

**Fig. 5:**
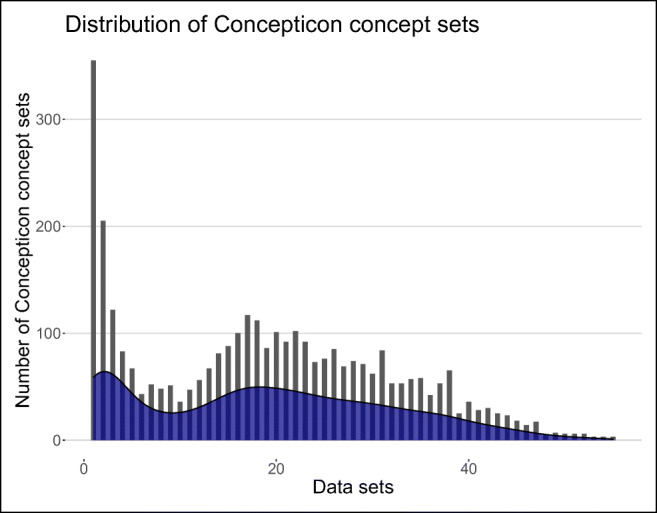
Distribution of Concepticon concept sets across the NoRaRe data sets. The *x*-axis gives the number of data sets in which the concept sets occur. The *y*-axis provides the number of Concepticon concept sets

The distribution of the Concepticon concept sets across the 98 data sets is illustrated in Fig. 5. The graph shows that most Concepticon concept sets occur in only a few data sets. Nevertheless, a large group of Concepticon concept sets is linked to 15 to 20 data sets (mean = 18.79). The most frequently occurring concept sets that are mapped to 64 up to 74 data sets are given in Table 3. Almost all of them belong to concepts representing concrete objects such as dog, eye, or bird. There is only one exception: white. In total, 3554 from 3743 Concepticon concept sets were linked to at least one NoRaRe data set.

**Table 3:** The 15 most common Concepticon concept sets occurring in 64 up to 74 NoRaRe data sets

Rank	ID	Concept set	Data sets
1	2009	dog	74
2	1248	eye	74
3	937	bird	73
4	1489	cloud	72
5	227	fish	71
6	1343	sun	71
7	1430	star	71
8	1223	heart	69
9	730	snake	67
10	906	tree	67
11	1221	nose	67
12	1394	bone	67
13	1247	ear	64
14	1297	leg	64
15	1335	white	64

The numbers reported in this section illustrate that the NoRaRe collection offers a wide range of data sets across multiple structural types such as numeric, categorical, and relational data. Although we restrict the number of words in a given data set to the number of currently available Concepticon concept sets (3743 in Version 2.4.0., List et al., 2020a), we provide the basis for comparing several words and concepts across a variety of languages (see Table 4).

**Table 4:** Subset of the NoRaRe data. The table gives information on the language for which the data was collected, the data type, the original item number, and the number of matches to the Concepticon concept sets

	Language	Types	Items	Matches
Norms				
Cai and Brysbaert (2010)	Chinese	frequency	99,123	1644
Ferrand et al., (2010)	French	reaction time	38,840	1372
Brysbaert et al., (2011)	German	frequency	190,500	1291
Cuetos et al., (2011)	Spanish	frequency	94,338	1088
Alonso et al., (2011)	Spanish	frequency	67,979	1016
Tsang et al., (2018)	Chinese	reaction time	25,156	827
Keuleers et al., (2010)	Dutch	frequency	437,503	640
González-Nosti et al., (2014)	Spanish	reaction time	2765	554
Mandera et al., (2015)	Polish	frequency	377,843	215
Ratings				
Lynott et al., (2020)	English	sensorimotor	40,000	2437
Brysbaert et al., (2019)	English	prevalence	62,000	2414
Kuperman et al., (2012)	English	age-of-acquisition	30,000	2351
Stadthagen-González et al., (2017)	Spanish	valence, arousal	14,031	932
Moors et al., (2013)	Dutch	age-of-acquisition, affective*	4300	444
Łuniewska et al., (2019)	Diverse	age-of-acquisition	299	284
Verheyen et al., (2020)	Dutch	age-of-acquisition, lexicosemantic, distributional, affective, concreteness, imageability	1000	206
Imbir (2016)	Polish	age-of-acquisition, affective, concreteness, imageability	4900	159
Kapucu et al., (2018)	Turkish	discrete emotions, affective, concreteness	2031	75
Relations				
Wu et al., (2020)	Global	core vocabulary	10,000	2460
Matisoff (2015)	Sino-Tibetan (Global)	etymology	6431	2159
Starostin (2000)	Diverse	sense relation	7095	2020
Rzymski et al., (2020)	Global	polysemy	1624	1624
Bond and Foster (2013)	English	WordNet	4960	1309
Dellert and Buch (2018)	Eurasian	basicness, stability	1016	955
Hill et al., (2015)	English	semantic similarity	999	524
Calude and Pagel (2011)	Diverse	stability, frequency	200	200
Baroni and Lenci (2011)	English	semantic similarity	200	140

*The term ‘affective’ summarizes different variables such as arousal, valence, and dominance

## Using NoRaRe: Case studies

The NoRaRe database is intended to facilitate cross-linguistic comparison of word and concept properties. In addition, the data enable researchers from psychology and linguistics to benefit from the different perspectives and results that are collected in each field. The first example of a study that uses the data in NoRaRe investigated word frequencies across English, German, and Chinese (Tjuka, 2020b). The study showed that the words occurring in the SUBTLEX corpora (Brysbaert & New, 2009; Brysbaert et al., 2011; Cai & Brysbaert, 2010) have more similar frequencies in closely related languages (i.e., English and German) than non-related languages (i.e., English and Chinese). Since the data in NoRaRe is already in a unified format and mapped to the Concepticon concept sets, a correlation between different properties can be easily performed. In the NoRaRe GitHub repository, we provide example scripts for Python and R so that data points across various data sets can be conveniently compared and plotted.^16^ The following case studies provide further examples for using the NoRaRe data and illustrate the validity of our approach.

### Case study 1: Replication of existing findings

In the first case study, we identified two similar data sets by using the labels of the word and concept properties in the NoRaRe database. The data sets were chosen to replicate existing results. The Concepticon includes more than 3000 concept sets. In studies with more or different items than concept sets in Concepticon, parts of the lists are not linked and the number of items is reduced. Therefore, we computed the correlation of three variables across two data sets to see whether the results are still significant.

The NoRaRe collection was filtered by variables to find lists with the same norms, ratings, and relations. We found several data sets that included ratings on arousal, valence, and dominance. To ensure that the data could be equally comparable, we identified the lists with ratings in the same language and the same rating scale. The search was easily carried out because each data set is labeled within the NoRaRe workflow. We selected two data sets for our study that provide ratings of English words on a nine-point scale for arousal, valence, and dominance: Warriner et al., (2013) and Scott et al., (2019). Both lists were linked with the automated workflow.

**Fig. 6:**
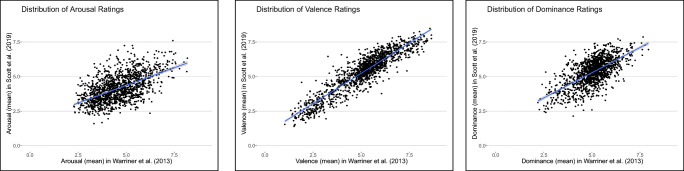
Distribution of the mean values for (*left*) arousal, (*middle*) valence, and (*right*) dominance in Warriner et al., (2013) and Scott et al., (2019) for 1397 Concepticon concept sets

The original list in Warriner et al., (2013) consisted of 13,915 English words. The mapping algorithm found 2067 links between the words in Warriner et al., (2013) and the Concepticon concept sets. In the case of Scott et al., (2019), the original list included 5500 words and there were 1459 matches with Concepticon concept sets. The overlap between both data sets in the NoRaRe database amounted to 1397 concept sets (the overlap between the original data sets was 4073 words). Table 5 shows the results of the correlations between the ratings for arousal, valence, and dominance in Warriner et al., (2013) and Scott et al., (2019). For each variable, the correlation (Pearson coefficients) was highly significant (*p*<.00001). The distribution of the ratings in Warriner et al., (2013) and Scott et al., (2019) across the nine-point scale for the 1397 Concepticon concept sets is illustrated in Fig. 6.

**Table 5:** Pearson coefficients for the variables arousal, valence, and dominance (see text). The values in parentheses indicate the original numbers, reported in Scott et al., (2019)

Overlap	Arousal	Valence	Dominance
1397 (4,073)	0.57 (0.62)	0.92 (0.93)	0.66 (0.69)

The additional information for each data set in the NoRaRe database facilitates access to relevant content. The labels of data sets provide the basis for an effortless comparison between the variety of data and allow fast identification of compatible variables across different data sets. The web application gives a clear display of the different data sets and more information about each property in a given data set can be found in the GitHub repository. The results of the correlation between Warriner et al., (2013) and Scott et al., (2019) replicated the findings reported in Scott et al., (2019). Thus, the reduction of the items due to the restricted number of Concepticon concept sets still ensures the comparability of data sets. This result may not hold for all data sets in the NoRaRe database, but the Concepticon resource is growing steadily and more concept sets are added with each release.

### Case study 2: Comparison of workflows

We used three workflows to link the data sets on norms, ratings, and relations to the Concepticon concept sets: *manual*, *automated*, and *semi-automated* (for a detailed description of the workflows, see Section “Workflows”). The main difference between the manual and both automated workflows lies in the check for accuracy of the mappings. In the manual workflow, the link between a given word and a Concepticon concept set is manually examined by a person who is familiar with the structure of Concepticon and a team of reviewers who discuss ambiguous cases. The automated workflow, on the other hand, uses an inherent rating system of the similarity between a word and the matches to the Concepticon concept sets without human intervention. In the second case study, we tested whether the links of both workflows are equal in their accuracy.

By searching the labels for the word and concept properties provided in the NoRaRe database, we identified similar data sets that include information for the same variable and language. In addition, lists which were linked with the manual versus automated workflow were considered.^17^ For the present study, we chose four data sets that offer ratings of English words on a seven-point scale for sensory modality (auditory, haptic, gustatory, olfactory, visual): Lynott and Connell (2013), Lynott and Connell (2009), Winter (2016), and Lynott et al., (2020). The former three were prepared with the manual workflow, the latter with the automated workflow.

**Fig. 7:**
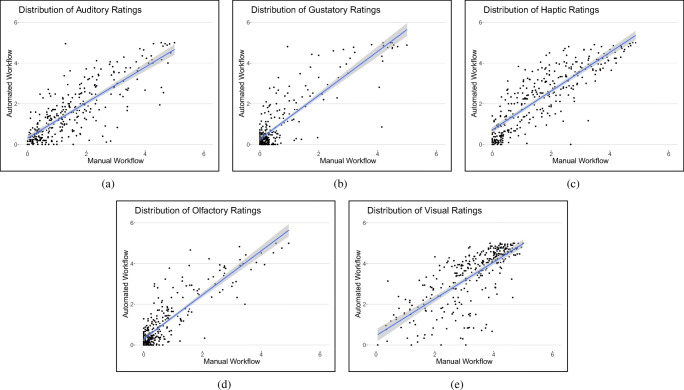
Distribution of the mean values for the five sensory modalities: **a** auditory, **b** gustatory, **c** haptic, **d** olfactory, and **e** visual in Lynott and Connell (2013), Lynott and Connell (2009), and Winter (2016) (manual workflow) and Lynott et al., (2020) (automated workflow) for 314 Concepticon concept sets

In the data of Lynott and Connell (2009) that consisted of 423 adjectives, of which 102 were linked to the Concepticon concept sets. From the original list in Lynott and Connell (2013), we linked 147 nouns to Concepticon concept sets from the original number of 400 items. In Winter (2016), 87 verbs of the 300-item list were linked to Concepticon concept sets. The original data set in Lynott et al., (2020) comprised 40,000 English words and the algorithm detected 2437 correspondences to Concepticon concept sets. The overlap between the manually prepared data sets and the automatically prepared data set was 314 Concepticon concept sets. The results of the correlation between the five sensory modality ratings are shown in Table 6. The correlations (Pearson coefficients) were highly significant (*p*<.00001) across all five variables. Figure 7 illustrates the distribution of the ratings in Lynott and Connell (2013), Lynott and Connell (2009), and Winter (2016) (manual workflow) and Lynott et al., (2020) (automated workflow) across a seven-point scale for the 314 Concepticon concept sets.

**Table 6:** Pearson coefficients for the sensorimotor variables auditory, gustatory, haptic, olfactory, and visual (see text). Abbreviations: AUD auditory; GUS gustatory; HAP haptic; OLF olfactory; VIS visual

Overlap	AUD	GUS	HAP	OLF	VIS
314	0.81	0.86	0.85	0.87	0.74

The convenient access to relevant information about the content of a given data set is provided by the data set identifiers in the NoRaRe database. We identified word lists that were prepared with the manual and automated workflow. The accuracy of the automated workflow seems to be as good as the manual workflow. The results indicate that both workflows can be equally applied for the different lists. Nevertheless, we will continue to add concept lists with the established Concepticon workflow because it ensures the quality of the mapping algorithm although the automated workflow is faster. This is especially important because word lists in psychology do not provide information on specific word meanings and in the manual workflow, we try to find the best link possible.

### Case study 3: Cross-linguistic comparison

The NoRaRe database is intended to facilitate cross-linguistic comparison. To illustrate the application of the data, we performed a pairwise comparison and correlation across the similarity ratings of 11 typologically diverse languages^18^ in Multi-SimLex (Vulić et al., 2020) and colexifications across several languages in CLICS^1^ (195 languages, List et al., 2013), CLICS^2^ (1220 languages, List et al., 2018), and CLICS^3^ (3156 languages, Rzymski et al., 2020). The study tested the degree to which similarities between words based on user ratings for a given language correlate with concepts based on colexifications.

Both data sets consist of pairs: On the one hand, the word pairs in Multi-SimLex were judged by native speakers who indicated how similar the words were on a scale of 0–6. The original list was based on English and was translated into 12 languages including Arabic, Chinese, Welsh, Hebrew, among others. Multi-SimLex was established as an alternative to similarity measures based on WordNet to improve models for distributional semantics and representation learning across multiple languages (Vulić et al., 2020). On the other hand, the data in CLICS stem from a cross-linguistic comparison of colexification patterns in several languages. The pairs in CLICS represent concepts that are colexified in diverse languages. The data is structured in form of a network with weighted degrees. The degrees indicate either *family weight* (i.e., in how many language families the colexification occurs) or *language weight* (i.e., in how many languages the colexification appears). For the present study, we used the family weight as the basis for the comparison.

The original data in Multi-SimLex contained 1888 pairs of which we linked 654 words to Concepticon concept sets (see List, 2021). The first version of CLICS^1^ (List et al., 2013) consisted of 1280 concept pairs, followed by 1105 concept pairs in version 2 (CLICS^2^, List et al., 2013) and 1624 concept pairs in version 3 (CLICS^3^, Rzymski et al., 2020). The overlap between the data sets was 252 Concepticon concept sets. First, we performed a correlation of the similarity ratings across the 11 languages in Multi-SimLex which resulted in a Pearson coefficient of *R* = .68 (strong correlation). Second, we compared the correlations between the similarity ratings in Multi-SimLex with the strength of family weights in the three versions of CLICS. The results showed that although the correlation with CLICS^1^ had a low positive Pearson coefficient of *R* = 0.34, the coefficients with the second and third versions of CLICS yielded negligible correlations (*R* = .25 and *R* = .27, respectively). An overview of the results is given in Table 7.

The study illustrates that the two data sets are only in part comparable. However, we expected that the comparison of the similarity ratings across the languages in Multi-SimLex based on the same word pairs would have resulted in an even higher correlation. The strong correlation indicates cross-linguistic similarities in the ratings, but a more detailed comparison may reveal language-specific rating patterns that could shed light on differences between the meanings of words across cultures. Interestingly, the low correlations with the three versions of CLICS demonstrate that the similarity ratings in Multi-SimLex seem to conflate different types of similarity, i.e., metaphor, metonymy, or meronomy. Since the data in CLICS include colexifications across languages, they indicate concepts that are commonly labeled with the same word which points to polysemy or homonymy. This shows that similarity needs to be more carefully defined in studies such as Multi-SimLex and the different relations need to be distinguished. The data in CLICS may even offer an alternative to Multi-SimLex for studies on semantic similarity. As shown by the third case study, we were able to successfully apply the data in NoRaRe for an extensive cross-linguistic comparison which emphasizes the usefulness of our approach.

## Discussion and conclusion

The available data describing word and concept properties are plentiful. Psychologists and linguists aggregate a great deal of valuable information, and both can benefit from the different perspectives taken in their respective fields. Research in psychology offers data on norms and ratings, whereas research in linguistics can contribute information about relations between words (recent studies show the importance of combining data from both fields: Calude and Pagel, 2011; Jackson et al., 2019). We set out to create a collection of these data and present the Database of Cross-Linguistic Norms, Ratings, and Relations for Words and Concepts (NoRaRe). The NoRaRe database is built on test-driven data curation, which implies workflows that connect the data to the Concepticon project. Since the aim of the Concepticon is to provide a reference catalog for comparable concepts, the data in NoRaRe can be compared across multiple languages. For convenient access, we provide a web application that presents an overview of the available data and allows for quick comparison.

The biggest challenge of our project was to transform a large number of different data sets so that they are comparable. Thus, we established three workflows to account for the different structures from small discrete lists with only a few words or concepts, larger lists with more than 2000 words or concepts, and online databases that can only be accessed via an API. The manual workflow for small lists used the process established for Concepticon concept lists and results in a hand-curated list that is reviewed by one of the Concepticon editors. In the automated workflow, large lists with up to several thousand words or concepts are automatically linked and prepared with little effort. The semi-automated workflow uses the advantages of the other two workflows in that the data are automatically linked but in case there are multiple options, they are selected manually. The replication of results from correlating similar data sets (case study 1) and the comparison of results from the manual versus automated workflow (case study 2) showed that our approach is valid and the data can be used in future studies. Especially for cross-linguistic studies, the NoRaRe database is the perfect starting point and properties such as frequencies can be compared easily across languages (as illustrated by case study 3 and Tjuka2020b).

**Table 7:** Pearson coefficients for pairwise comparison across languages for Multi-SimLex (Vulić et al., 2020), CLICS^1^ (List et al., 2013), CLICS^2^ (List et al., 2018), and CLICS^3^ (Rzymski et al., 2020)

Overlap (pairs)	Multi-SimLex	CLICS^1^	CLICS^2^	CLICS^3^
252	0.68	0.34	0.25	0.27

Yet, there are also limitations to our approach. Since the Concepticon consists of a limited number of concepts that are hand-curated, large-scale studies with over 3000 items cannot be performed. Although the Concepticon is growing steadily, it is not intended to replace crowd-sourced studies that use Amazon Mechanical Turk or the like. The Concepticon concept sets need to withstand the scrutiny of expert linguists and the feedback of the community so that it is constantly improved. The Concepticon editors are the curators of the links between elicitation glosses to a given concept set. This comes with a huge responsibility and requires intricate knowledge about the structure of the database. The manual workflow allows us to integrate quality control for each link to a given concept set in our review process (Tjuka, 2021). Nevertheless, if a mapping is ambiguous, we reserve the right to unmap (i.e., delete the mapping). The result of the manual workflow improves the mapping algorithm used for the automated workflow. The data sets prepared with the automated workflow are stored in a separate repository so that they do not influence the quality of the algorithm. As shown in case study 2, the links made by the automated workflow are comparable with the hand-curated mapping in Concepticon. However, large data sets can have thousands of possible links, so mismatches cannot be ruled out (for a detailed discussion about individual mappings, see Tjuka, 2020b).

A clear advantage of our approach is that the data are infinitely extendable. Although databases for word properties are available for researchers to use freely and some of them offer a broad range of content (e.g., Baayen et al., 1996; Heister et al., 2011; Buchanan et al., 2019a), they are often not regularly updated or left alone after the publication of the associated article (e.g., Winter et al., 2017; Wilson, 1988). The Concepticon project exists since 2016 and is constantly growing. It is also a seedbed for new features that combine the data in new ways, for example, to compile colexification networks (List et al., 2018; Rzymski et al., 2020). The NoRaRe is another example of how the Concepticon can be used as a reference catalog to bring together data sets of different fields so that new questions can be answered. Another avenue would be to add neurocognitive resources that offer norms based on brain imaging (e.g., Vassallo et al., 2018; Stehwien et al., 2020) to NoRaRe.^19^ The projects are all open-ended in that they will continue to be updated, improved, and extended. This is possible due to the test-driven data curation workflows, publicly curating the data on GitHub, and a continuously growing community that uses our tools.^20^ While we provide workflows to standardize data sets, we hope that our description of the fair data principles (Wilkinson et al., 2016) inspires researchers to prepare their lists in a more sufficient way. With the newly developed automated and semi-automated workflows, new data sets can easily be added and the test-driven data curation guarantees consistency of the data. The quality checks provided by the integrated tests in the Python libraries pyconcepticon and pynorare are supported by the review process on GitHub.

The NoRaRe database facilitates a comparison of word and concept properties across diverse languages. New cross-linguistic resources, for instance, age-of-acquisition ratings across a diverse set of languages (Łuniewska et al., 2016; Łuniewska et al., 2019) or Multi-SimLex (Vulić et al., 2020), that was added to Concepticon recently (List, 2021), extend the available mapping languages in Concepticon. Apart from data on languages spoken by many people, they additionally offer ratings for smaller languages: Estonian, Gaelic, Icelandic, Welsh, among others. In the future, we aim to add extensive word lists based on the Intercontinental Dictionary Series (Key and Comrie, 2016; List, 2020) in other languages, for example, Italian and Vietnamese, to widen the cross-linguistic scope of Concepticon further. Case study 3 was a first illustration of the cross-linguistic comparisons that are made possible with the NoRaRe data. It revealed that when comparing similarity ratings in 11 different languages in Multi-SimLex, the agreement for the same word pairs is not as high as expected. In addition, the comparison with the three versions of CLICS which include colexifications across languages was even lower, indicating differences in the types of similarities stored in the two resources. It would be interesting to compare both data sets also with WordNet in the future because Multi-SimLex, as well as CLICS, offer additional measures that further illustrate the different relations between concepts (i.e., association, polysemy, homonymy).

Since the Concepticon project is a multilingual resource, it needs to be distinguished from Linked Data approaches like WordNet (Fellbaum, 1998; Princeton University, 2010). Whereas WordNet provides the concrete meaning of words in a given language, the Concepticon project aims to offer a standardization for concepts across languages. The Concepticon can be seen as a bridge between the linguistics community and the Linked Data community. The value of our approach is that it allows further applications of cross-linguistic semantic comparison based on hand-curated data from expert linguists. In addition, researchers using Linked Data approaches can benefit from the historical perspective that our resources offer.

The web application for the NoRaRe database offers researchers with a concise overview of the available data sets. The metadata for each data set includes labels for the different data types (norms, ratings, and relations), as well as tags for each data point in a given list that give information on how the data was collected, for instance, which scale was used in a rating study. All data sets in NoRaRe are structured the same ways so that they can be easily compared, which is especially important for filling in gaps or finding out who has done similar research that you were planning to do. For now, we have selected data sets that are either relevant to our future research, randomly chosen, or suggested by the reviewers of the present article. However, there is nothing to stop us from adding data on word and concept properties that were not included in the second version of NoRaRe. Similar to the Concepticon project, we envisage the NoRaRe database to be a community project and we encourage researchers to point out data sets that should be added to NoRaRe or contribute their own data set via the GitHub repository.

Linking norms, ratings, and relations of words and concepts across multiple languages is a contribution to interdisciplinary research in psychology and linguistics. We are confident that our efforts will be fruitful and contribute to understanding language through a cross-linguistic perspective. Our data curation is test-driven and provides a framework that can be extended indefinitely. We invite other researchers to test and use our database to answer their research questions.

## Electronic supplementary material

Below is the link to the electronic supplementary material.

(PDF 85.8 KB)

## Data Availability

The database of Cross-Linguistic Norms, Ratings, and Relations for Words and Concepts (NoRaRe) presented in this article is curated on GitHub (https://doi.org/https://github.com/concepticon/norare-data) and archived with Zenodo (10.5281/zenodo.3957680). The Concepticon database (List et al., 2020a) is also curated on GitHub (https://github.com/concepticon/concepticon-data) and archived on Zenodo (10.5281/zenodo.596412). R-Scripts that were used to produce the plots for the three case studies are available from the NoRaRe collection (on GitHub concepticon/norare-data, folder examples). The GitHub repository also provides detailed instructions on the installation of the curation software and the details of the data curation process. The Python package used for the data curation workflow can also be found on GitHub (https://github.com/concepticon/pynorare). The pynorare package is stored on Zenodo (10.5281/zenodo.3946713) as well as PyPi (https://pypi.org/project/pynorare/). For convenient access to the NoRaRe database, we offer a web application: https://digling.org/norare/.

## Footnotes

1 The database curated by the Concepticon project can be accessed via a web application under the following link: https://concepticon.clld.org

2 Note that ratings are also described as norms in the literature (e.g., Scott et al., 2019). Thus, the terms ‘norms’ and ‘ratings’ are to an extent used interchangeably and there seems no apparent categorical distinction. For our purposes, we decided to make a clear distinction since it allows a better overview of the data.

3 The term was first introduced by François (2008). It is a cover term for polysemy and homonymy. Thus, colexification refers to those cases in which the same word in a given language is used to express two or more concepts, such as Russian *ruka*, Hausa *hannu*, or Vietnamese *tay* all denoting ‘hand’ and ‘arm’.

4 See https://zenodo.org

5 See https://osf.io

6 The Wikidata project is available under the following link: https://www.wikidata.org/

7 BabelNet is available online: https://babelnet.org/

8 The OmegaWiki project can be accessed with the following link: https://omegawiki.org

9 The data can be accessed under the following link: https://digling.org/norare/

10 The file norare.tsv includes all relevant information about each individual property offered in a data set and together with the metadata of each data set in concept_set_meta.tsv builds the foundation of the web application. Both files can be accessed in the GitHub repository: https://github.com/concepticon/norare-data/tree/v0.2/norare.tsv and https://github.com/concepticon/norare-data/tree/v0.2/concept_set_meta.tsv.

11 More information can be found here: https://clld.org/

12 The Concepticon resource already included data on norms and relations in earlier versions (List et al., 2016). We added those nine data sets to the NoRaRe collection.

13 The full list of Concepticon releases can be found here: https://github.com/concepticon/concepticon-data/releases

14 The repository is available here: https://github.com/concepticon/norare-data

15 The full list of NoRaRe releases can be found here: https://github.com/concepticon/norare-data/releases

16 The example scripts including the correlations for the case studies are available here: https://github.com/concepticon/norare-data/tree/v0.2/examples

17 The identifier for manually prepared data sets has the format ‘Author-Year-Number of items’ (e.g., ‘Lynott-2013-400’), whereas the identifier for automatically prepared data sets is structured in a different format: ‘Author-Year-Main property’ (e.g., ‘Lynott-2020-Sensorimotor’).

18 Note that we did not include the data on Kiswahili since our quality check revealed inconsistencies in the translations.

19 We thank an anonymous reviewer for pointing out these references.

20 For a list of Concepticon contributors, see https://concepticon.clld.org/contributors
