# Supplementary material for: Linking norms, ratings, and relations of words and concepts across multiple language varieties
Source: Behav Res Methods. 2021 Aug 6;54(2):864–84. doi: 10.3758/s13428-021-01650-1 (PMC9046307; doi:10.3758/s13428-021-01650-1)
Supplement: Supplementary file 1 — (PDF 85.8 KB) [file 13428_2021_1650_MOESM1_ESM.pdf]

List of Data Sets in the NoRaRe database

Annika Tjuka

March 30th, 2021

Table 1: A complete list of the data sets that are included in the NoRaRe database (Version 0.2, Tjuka et al., 2021) in no particular order. The list was created on March 30th, 2021. It includes references to the data sets, the investigated language, NoRaRe tags, and the matches to the Concepticon database (Version 2.4.0., List et al., 2020). Since we update our databases regularly, the latest list of data sets and additional details can be found in the GitHub repository: `https://github.com/concepticon/norare-data`

**No.**	**Author**	**Language**	**Tags**	**Concepticon Matches**
1	Bond and Foster (2013)	English	relations	1309
2	Alonso et al. (2015)	Spanish	ratings	836
3	Brysbaert and New (2009)	English	norms	2329
4	Brysbaert et al. (2011)	German	norms	1291
5	Brysbaert et al. (2014)	English	ratings	2344
6	Brysbaert et al. (2019)	English	ratings	2414
7	Cai and Brysbaert (2010)	Chinese	norms	1644
8	Cuetos et al. (2011)	Spanish	norms	1088
9	Desrochers and Thompson (2009)	French	ratings	567
10	Engelthaler and Hills (2018)	English	ratings	1334
11	Juhasz and Yap (2013)	English	ratings	1690
12	Keuleers et al. (2010)	Dutch	norms	640
13	Kuperman et al. (2012)	English	ratings	2351
14	Riegel et al. (2015)	Polish	ratings	98
15	Scott et al. (2019)	English	ratings	1459
16	Stadthagen-González et al. (2017)	Spanish	ratings	932
17	S. A. Starostin (2000)	English	relations	2020
18	Warriner et al. (2013)	English	ratings	2067
19	Cortese and Khanna (2008)	English	ratings	1163
20	Keuleers et al. (2012)	English	norms	2119
21	Ferrand et al. (2010)	French	norms	1372
22	González-Nosti et al. (2014)	Spanish	norms, ratings	554
23	Tsang et al. (2018)	Chinese	norms	827
24	Keuleers et al. (2015)	Dutch	ratings	644
25	Stadthagen-González et al. (2018)	Spanish	ratings	467
26	Alonso et al. (2016)	Spanish	ratings	294
27	Imbir (2016)	Polish	ratings	159
28	Ferré et al. (2017)	Spanish	ratings	387
29	Wierzba et al. (2015)	Polish	ratings	98
30	Alonso et al. (2011)	Spanish	norms	1016
31	Lynott et al. (2020)	English	ratings	2437
32	Kapucu et al. (2018)	Turkish	ratings	75
33	Briesemeister et al. (2011)	German	ratings	401

34	Mandera et al. (2015)	Polish	norms	215
35	Moors et al. (2013)	Dutch	ratings	444
36	Wu et al. (2020)	Global	relations	2460
37	Mohammad (2018a)	English	ratings	2173
38	Mohammad (2018b)	English	ratings	741
39	Clark and Paivio (2004)	English	ratings	758
40	Abdaoui et al. (2017)	French	relations	1111
41	Matisoff (2015)	Sino-Tibetan (Global)	relations	2159
42	Kiss et al. (1973)	English	relations	1376
43	Izura et al. (2005)	Spanish	norms, ratings	251
44	Winter (2016)	English	ratings	88
45	Hill et al. (2015)	English	relations	524
46	Lewis and Frank (2016)	English	ratings	148
47	Rzymski et al. (2020)	Global	relations	1624
48	Xiao and Treiman (2012)	Chinese	norms, ratings	158
49	Yao et al. (2017)	English	ratings	288
50	Pagel et al. (2007)	Diverse	relations	200
51	Łuniewska et al. (2016)	Diverse	ratings	283
52	Schroeder et al. (2012)	German	ratings	246
53	Dellert and Buch (2018)	Eurasian	relations	955
54	Verheyen et al. (2020)	Dutch	ratings, relations	206
55	Díez-Álamo et al. (2018)	Spanish	ratings	420
56	Monnier and Syssau (2014)	French	ratings	582
57	Gampe et al. (2017)	English	ratings	48
58	Lynott and Connell (2013)	English	ratings	148
59	Lynott and Connell (2009)	English	ratings	100
60	Desrochers et al. (2010)	Spanish	ratings	123
61	Pagel and Meade (2018)	Diverse	relations	200
62	Baroni and Lenci (2011)	English	relations	140
63	Maciejewski and Klepousniotou (2016)	English	ratings	64
64	Łuniewska et al. (2019)	Diverse	ratings	284
65	Calude and Pagel (2011)	Diverse	basic	200
66	Haspelmath and Tadmor (2009)	Diverse	relations	1459
67	Wikimedia (2020)	English	relations	1194
68	Merriam-Webster (2020)	English	relations	36
69	OmegaWiki (2020)	Diverse	relations	2070
70	Aristar-Dry (2015)	Diverse	relations	1344
71	BabelNet (2020)	English	relations	1127
72	Crepaldi et al. (2015)	Italian	norms	261
73	van Heuven et al. (2014)	English	norms	2448
74	Medler et al. (2005)	English	ratings	689
75	Gilhooly and Logie (1980)	English	ratings	630
76	Vulić et al. (2020)	Diverse	ratings	869
77	Vejdemo and Hörberg (2016)	Diverse	ranked, ratings	167
78	Numerals (2020)	Global	relations	161
79	S. Starostin (2007)	Global	ranked	110
80	Tadmor (2009)	Global	ranked	100
81	Dyen (1964)	Malayo-Polynesian	ranked	196
82	Dyen (1964)	Indo-European	ranked	153
83	Thomas (1960)	Mon-Khmer	ranked	167
84	Wu et al. (2020)	Global	relations	2460
85	Pozdniakov (2014)	Atlantic	ranked	100
86	Carling et al. (2019)	Eurasian	lolo, ranked	99
87	Zalizniak et al. (2020)	Global	norms, relations	1469

88	Scheible and Schulte im Walde (2014)	German	ratings	408
89	Lapesa et al. (2014)	English	ratings	222
90	Vergallito et al. (2020)	Italian	ratings	508
91	Johansson et al. (2020)	Global	basic	285
92	Speed and Majid (2017)	Dutch	ratings	250
93	Chen et al. (2019)	Chinese	ratings	86
94	Chen et al. (2019)	Chinese	ratings	20
95	Miklashevsky (2018)	Russian	ratings	253
96	Morucci et al. (2019)	Italian	ratings	123
97	Blomberg et al. (2020)	Swedish	ratings	83
98	Swadesh (1955)	Global	ranked	215

References

Abdaoui, A., Azé, J., Bringay, S., & Poncelet, P. (2017). FEEL: French Expanded Emotion Lexicon. Language resources and evaluation. *Language Resources and Evaluation*, *51*(3), 833–855. doi: 10.1007/s10579-016-9364-5

Alonso, M. Á., Díez, E., & Fernandez, A. (2016). Subjective age-of-acquisition norms for 4,640 verbs in Spanish. *Behavior Research Methods*, *48*(4), 1337–1342. doi: 10.3758/s13428-015-0675-z

Alonso, M. Á., Fernandez, A., & Díez, E. (2011). Oral frequency norms for 67,979 Spanish words. *Behavior Research Methods*, *43*(2), 449–458. doi: 10.3758/s13428-011-0062-3

Alonso, M. Á., Fernandez, A., & Díez, E. (2015). Subjective age-of-acquisition norms for 7,039 Spanish words. *Behavior Research Methods*, *47*(1), 268–274. doi: 10.3758/s13428-014-0454-2

Aristar-Dry, H. (2015). *Lexicon Enhancement via the GOLD Ontology.* Retrieved from `https://lego.linguistlist.org/`

BabelNet. (2020). *BabelNet. Search, translate, learn.* Retrieved from `https://babelnet.org`

Baroni, M., & Lenci, A. (2011). *BLESS: Baroni & Lenci's evaluation of semantic similarity.* Retrieved from `https://sites.google.com/site/geometricalmodels/shared-evaluation`

Blomberg, F., Roll, M., Frid, J., Lindgren, M., & Horne, M. (2020). The role of affective meaning, semantic associates, and orthographic neighbours in modulating the N400 in single words. *The Mental Lexicon*, *15*(2), 161–188. doi: 10.1075/ml.19021.blo

Bond, F., & Foster, R. (2013). Linking and extending an Open Multilingual WordNet. In H. Schuetze, P. Fung, & M. Poesio (Eds.), *Proceedings of the 51st Annual Meeting of the Association for Computational Linguistics (Volume 1: Long Papers)* (pp. 1352–1362). Sofia, Bulgaria: Association for Computational Linguistics. Retrieved from `http://compling.hss.ntu.edu.sg/omw/summx.html`

Briesemeister, B. B., Kuchinke, L., & Jacobs, A. M. (2011). Discrete emotion norms for nouns: Berlin affective word list (DENN-BAWL). *Behavior Research Methods*, *43*(2), 441–448. doi: 10.3758/s13428-011-0059-y

Brysbaert, M., Buchmeier, M., Conrad, M., Jacobs, A. M., Bölte, J., & Böhl, A. (2011). The word frequency effect: A review of recent developments and implications for the choice of frequency estimates in German. *Experimental Psychology*, *58*(5), 412–424. doi: 10.1027/1618-3169/a000123

Brysbaert, M., Mandera, P., McCormick, S. F., & Keuleers, E. (2019). Word prevalence norms for 62,000 English lemmas. *Behavior Research Methods*, *51*(2), 467–479. doi: 10.3758/s13428-018-1077-9

Brysbaert, M., & New, B. (2009). Moving beyond Kučera and Francis: A critical evaluation of current word frequency norms and the introduction of a new and improved word frequency measure for American English. *Behavior Research Methods*, *41*(4), 977–990. doi: 10.3758/BRM.41.4.977

Brysbaert, M., Warriner, A., & Kuperman, V. (2014). Concreteness ratings for 40 thousand generally known English word lemmas. *Behavior Research Methods*, *46*(3), 904–911. doi: 10.3758/s13428-013-0403-5

Cai, Q., & Brysbaert, M. (2010). SUBTLEX-CH: Chinese word and character frequencies based on film subtitles. *PLoS ONE*, *5*(6), 1–8. doi: 10.1371/journal.pone.0010729

Calude, A. S., & Pagel, M. (2011). How do we use language? Shared patterns in the frequency of word use across 17 world languages. *Philosophical Transactions of the Royal Society B: Biological Sciences*, *366*(1567), 1101–1107. doi: 10.1098/rstb.2010.0315

Carling, G., Cronhamn, S., Farren, R., Aliyev, E., & Frid, J. (2019, 10). The causality of borrowing: Lexical loans in Eurasian languages. *PLoS ONE*, *14*(10), 1-33. Retrieved from `https://doi.org/10.1371/journal.pone`

.0223588 doi: 10.1371/journal.pone.0223588
Chen, I.-H., Zhao, Q., Long, Y., Lu, Q., & Huang, C.-R. (2019). Mandarin Chinese modality exclusivity norms. *PLoS ONE*, *14*(2), 1-18.
Clark, J. M., & Paivio, A. (2004). Extensions of the Paivio, Yuille, and Madigan (1968) norms. *Behavior Research Methods*, *36*(3), 371–383. doi: 10.3758/BF03195584
Cortese, M. J., & Khanna, M. M. (2008). Age of acquisition ratings for 3,000 monosyllabic words. *Behavior Research Methods*, *40*(3), 791–794. doi: 10.3758/BRM.40.3.791
Crepaldi, D., Amenta, S., Pawel, M., Keuleers, E., & Brysbaert, M. (2015). *SUBTLEX-IT. Subtitle-based word frequency estimates for Italian.* Rovereto. (Talk presented at Proceedings of the Annual Meeting of the Italian Association For Experimental Psychology)
Cuetos, F., Glez-Nosti, M., Barbón, A., & Brysbaert, M. (2011). SUBTLEX-ESP: Spanish word frequencies based on film subtitles. *Psicológica*, *33*(2), 133–143.
Dellert, J., & Buch, A. (2018). A new approach to concept basicness and stability as a window to the robustness of concept list rankings. *Language Dynamics and Change*, *8*(2), 157–181. doi: 10.1163/22105832-00802001
Desrochers, A., Liceras, J. M., Fernandez-Fuertes, R., & Thompson, G. L. (2010). Subjective frequency norms for 330 Spanish simple and compound words. *Behavior Research Methods*, *42*(1), 109–117. doi: 10.3758/BRM.42.1.109
Desrochers, A., & Thompson, G. L. (2009). Subjective frequency and imageability ratings for 3,600 French nouns. *Behavior Research Methods*, *41*(2), 546–557. doi: 10.3758/BRM.41.2.546
Díez-Álamo, A. M., Díez, E., Alonso, M. Á., Vargas, C. A., & Fernandez, A. (2018). Normative ratings for perceptual and motor attributes of 750 object concepts in Spanish. *Behavior Research Methods*, *50*(4), 1632–1644. doi: 10.3758/s13428-017-0970-y
Dyen, I. (1964). On the validity of comparative lexicostatistics. In *Proceedings of the international congress of linguistics* (p. 238-252). Cambridge: Sijthoff.
Engelthaler, T., & Hills, T. T. (2018). Humor norms for 4,997 English words. *Behavior Research Methods*, *50*(3), 1116–1124. doi: 10.3758/s13428-017-0930-6
Ferrand, L., New, B., Brysbaert, M., Keuleers, E., Bonin, P., Méot, A., … Pallier, C. (2010). The French Lexicon Project: Lexical decision data for 38,840 French words and 38,840 pseudowords. *Behavior Research Methods*, *42*(2), 488–496. doi: 10.3758/BRM.42.2.488
Ferré, P., Guasch, M., Martínez-García, N., Fraga, I., & Hinojosa, J. A. (2017). Moved by words: Affective ratings for a set of 2,266 Spanish words in five discrete emotion categories. *Behavior Research Methods*, *49*(3), 1082–1094. doi: 10.3758/s13428-016-0768-3
Gampe, A., Kurthen, I., & Daum, M. M. (2017). BILEX: A new tool measuring bilingual children's lexicons and translational equivalents. *First Language*, *38*(3), 263–283. doi: 10.1177/0142723717736450
Gilhooly, K. J., & Logie, R. H. (1980). Age-of-acquisition, imagery, concreteness, familiarity, and ambiguity measures for 1,944 words. *Behavior Research Methods & Instrumentation*, *12*(4), 395–427. doi: 10.3758/BF03201693
González-Nosti, M., Barbón, A., Rodríguez-Ferreiro, J., & Cuetos, F. (2014). Effects of the psycholinguistic variables on the lexical decision task in Spanish: A study with 2,765 words. *Behavior Research Methods*, *46*(2), 517–525. doi: 10.3758/s13428-013-0383-5
Haspelmath, M., & Tadmor, U. (2009). *Loanwords in the world's languages. A comparative handbook.* Berlin/New York: Walter de Gruyter.
Hill, F., Reichart, R., & Korhonen, A. (2015). SimLex-999: Evaluating semantic models with (genuine) similarity estimation. *Computational Linguistics*, *41*(4), 665–695. doi: 10.1162/COLI_a_00237
Imbir, K. K. (2016). Affective norms for 4900 Polish words reload (ANPW_R): Assessments for valence, arousal, dominance, origin, significance, concreteness, imageability, and age of acquisition. *Frontiers in Psychology*, *7*, 1–18. doi: 10.3389/fpsyg.2016.01081
Izura, C., Hernández-Muñoz, N., & Ellis, A. W. (2005). Category norms for 500 Spanish words in five semantic categories. *Behavior Research Methods*, *37*(3), 385–397. doi: 10.3758/BF03192708
Johansson, N. E., Anikin, A., Carling, G., & Holmer, A. (2020). The typology of sound symbolism: Defining macro-concepts via their semantic and phonetic features. *Linguistic Typology*, *24*(2), 253-310. doi: 10.1515/lingty-2020-2034
Juhasz, B. J., & Yap, M. J. (2013). Sensory experience ratings for over 5,000 mono- and disyllabic words. *Behavior Research Methods*, *45*(1), 160–168. doi: 10.3758/s13428-012-0242-9
Kapucu, A., Kılıç, A., Özkılıç, Y., & Sarıbaz, B. (2018). Turkish emotional word norms for arousal, valence, and discrete emotion categories. *Psychological Reports*, *0*(0), 1–22. doi: 10.1177/0033294118814722

Keuleers, E., Brysbaert, M., & New, B. (2010). SUBTLEX-NL: A new measure for Dutch word frequency based on film subtitles. *Behavior Research Methods*, *42*(3), 643–650. doi: 10.3758/BRM.42.3.643

Keuleers, E., Lacey, P., Rastle, K., & Brysbaert, M. (2012). The British Lexicon Project: Lexical decision data for 28,730 monosyllabic and disyllabic English words. *Behavior Research Methods*, *44*(1), 287–304. doi: 10.3758/s13428-011-0118-4

Keuleers, E., Stevens, M., Mandera, P., & Brysbaert, M. (2015). Word knowledge in the crowd: Measuring vocabulary size and word prevalence in a massive online experiment. *The Quarterly Journal of Experimental Psychology*, *68*(8), 1665–1692. doi: 10.1080/17470218.2015.1022560

Kiss, G. R., Armstrong, C., & Milroy, R. (1973). An associative thesaurus of English and its computer analysis. In A. J. Aitken, R. W. Bailey, & N. Hamilton-Smith (Eds.), *The computer and literary studies.* Edinburgh, UK: Edinburgh University Press.

Kuperman, V., Stadthagen-González, H., & Brysbaert, M. (2012). Age-of-acquisition ratings for 30,000 English words. *Behavior Research Methods*, *44*(4), 978–990. doi: 10.3758/s13428-012-0210-4

Lapesa, G., Schulte im Walde, S., & Evert, S. (2014). *Judging paradigmatic relations: A new collection of English ratings.* Poster presented at the 20th Architectures and Mechanisms for Natural Language Processing Conference (AMLaP).

Lewis, M. L., & Frank, M. C. (2016). The length of words reflects their conceptual complexity. *Cognition*, *153*, 182–195. doi: 10.1016/j.cognition.2016.04.003

List, J.-M., Rzymski, C., Greenhill, S. J., Schweikhard, N. E., Pianykh, K., Tjuka, A., … Forkel, R. (2020). *Concepticon. A resource for the linking of concept lists (Version 2.4.0).* Jena: Max Planck Institute for the Science of Human History. doi: 10.5281/zenodo.4162002

Łuniewska, M., Haman, E., Armon-Lotem, S., Etenkowski, B., Southwood, F., Anđelković, D., … Ünal-Logacev, Ö. (2016). Ratings of age of acquisition of 299 words across 25 languages: Is there a cross-linguistic order of words? *Behavior Research Methods*, *48*(3), 1154–1177. doi: 10.3758/s13428-015-0636-6

Łuniewska, M., Wodniecka, Z., Miller, C. A., Smolík, F., Butcher, M., Chondrogianni, V., … Haman, E. (2019). Age of acquisition of 299 words in seven languages: American English, Czech, Gaelic, Lebanese Arabic, Malay, Persian and Western Armenian. *Plos ONE*, *14*(8), 1–19. doi: 10.1371/journal.pone.0220611

Lynott, D., & Connell, L. (2009). Modality exclusivity norms for 423 object properties. *Behavior Research Methods*, *41*(2), 558–564. doi: 10.3758/BRM.41.2.558

Lynott, D., & Connell, L. (2013). Modality exclusivity norms for 400 nouns: The relationship between perceptual experience and surface word form. *Behavior Research Methods*, *45*(2), 516–526. doi: 10.3758/s13428-012-0267-0

Lynott, D., Connell, L., Brysbaert, M., Brand, J., & Carney, J. (2020). The Lancaster Sensorimotor Norms: multidimensional measures of perceptual and action strength for 40,000 English words. *Behavior Research Methods*, *52*, 1271–1291. doi: 10.3758/s13428-019-01316-z

Maciejewski, G., & Klepousniotou, E. (2016). Relative meaning frequencies for 100 homonyms: British eDom norms. *Journal of Open Psychology Data*, *4*(1), 1–5. doi: 10.5334/jopd.28

Mandera, P., Keuleers, E., Wodniecka, Z., & Brysbaert, M. (2015). SUBTLEX-PL: Subtitle-based word frequency estimates for Polish. *Behavior Research Methods*, *47*(2), 471–483. doi: 10.3758/s13428-014-0489-4

Matisoff, J. A. (2015). *The Sino-Tibetan Etymological Dictionary and Thesaurus.* Department of Linguistics at the University of California, Berkeley. Retrieved from `https://stedt.berkeley.edu/`

Medler, D. A., Arnoldussen, A., Binder, J. R., & Seidenberg, M. S. (2005). *Wisconsin Perceptual Attribute Rating Database* [Database]. Retrieved 2021-02-24, from `http://www.neuro.mcw.edu/ratings/`

Merriam-Webster. (2020). *Merriam-Webster Dictionary.* Retrieved from `https://www.merriam-webster.com/`

Miklashevsky, A. (2018). Perceptual experience norms for 506 Russian nouns: Modality rating, spatial localization, manipulability, imageability and other variables. *Journal of Psycholinguistic Research*, *47*(3), 641-661.

Mohammad, S. M. (2018a). Obtaining reliable human ratings of valence, arousal, and dominance for 20,000 English words. In I. Gurevych & Y. Miyao (Eds.), *Proceedings of the 56th Annual Meeting of the Association for Computational Linguistics (Volume 1: Long Papers)* (pp. 174–184). Melbourne, Australia: Association for Computational Linguistics.

Mohammad, S. M. (2018b). Word Affect Intensities. In I. Gurevych & Y. Miyao (Eds.), *Proceedings of the Eleventh International Conference on Language Resources and Evaluation (LREC 2018)* (pp. 174–184). Melbourne, Australia: Association for Computational Linguistics.

Monnier, C., & Syssau, A. (2014). Affective norms for French words (FAN). *Behavior Research Methods*, *46*(4),

1128–1137. doi: 10.3758/s13428-013-0431-1

Moors, A., De Houwer, J., Hermans, D., Wanmaker, S., Van Schie, K., Van Harmelen, A.-L., ... Brysbaert, M. (2013). Norms of valence, arousal, dominance, and age of acquisition for 4,300 Dutch words. *Behavior Research Methods*, *45*(1), 169–177. doi: 10.3758/s13428-012-0243-8

Morucci, P., Bottini, R., & Crepaldi, D. (2019). Augmented modality exclusivity norms for concrete and abstract Italian property words. *Journal of Cognition*, *2*(1).

Numerals. (2020). *Mapping from concepticon concept sets specifying numerals to integer numbers.* Retrieved from `https://concepticon.clld.org/`

OmegaWiki, F. (2020). *OmegaWiki. A dictionary in all languages.* Retrieved from `https://www.omegawiki.org`

Pagel, M. D., Atkinson, Q. D., & Meade, A. (2007). Frequency of word-use predicts rates of lexical evolution throughout Indo-European history. *Nature*, *449*, 717–721. doi: 10.1038/nature06176

Pagel, M. D., & Meade, A. (2018). The deep history of the number words. *Philosophical Transactions of the Royal Society B*, *373*(1740), 1–9. doi: 10.1098/rstb.2016.0517

Pozdniakov, K. (2014). O poroge rodstva i indekse stabil'nosti v bazisnoj leksike pri massovom sravnenii: Atlantičeskie jazyki[On the threshold of relationship and the "stability index" of basic lexicon in mass comparison: Atlantic languages]. *Journal of Language Relationship*, *11*, 187-237. Retrieved from `http://jolr.ru/files/(144)jlr2014-11(187-237).pdf`

Riegel, M., Wierzba, M., Wypych, M., Żurawski, Ł., Jednoróg, K., Grabowska, A., & Marchewka, A. (2015). Nencki affective word list (NAWL): the cultural adaptation of the Berlin affective word list-reloaded (BAWL-R) for Polish. *Behavior Research Methods*, *47*(4), 1222–1236. doi: 10.3758/s13428-014-0552-1

Rzymski, C., Tresoldi, T., Greenhill, S. J., Wu, M.-S., Schweikhard, N. E., Koptjevskaja-Tamm, M., ... List, J.-M. (2020). The Database of Cross-Linguistic Colexifications, reproducible analysis of cross-linguistic polysemies. *Scientific Data*, *7*(1), 1–12. doi: 10.1038/s41597-019-0341-x

Scheible, S., & Schulte im Walde, S. (2014). A Database of Paradigmatic Semantic Relation Pairs for German Nouns, Verbs, and Adjectives. In J. Baptista et al. (Eds.), *Proceedings of Workshop on Lexical and Grammatical Resources for Language Processing* (pp. 111–119). Dublin, Ireland: Association for Computational Linguistics and Dublin City University. doi: 10.3115/v1/W14-5814

Schroeder, A., Gemballa, T., Ruppin, S., & Wartenburger, I. (2012). German norms for semantic typicality, age of acquisition, and concept familiarity. *Behavior Research Methods*, *44*(2), 380–394. doi: 10.3758/s13428-011-0164-y

Scott, G. G., Keitel, A., Becirspahic, M., Yao, B., & Sereno, S. C. (2019). The Glasgow Norms: Ratings of 5,500 words on nine scales. *Behavior Research Methods*, *51*(3), 1258–1270. doi: 10.3758/s13428-018-1099-3

Speed, L. J., & Majid, A. (2017). Dutch modality exclusivity norms: Simulating perceptual modality in space. *Behavior Research Methods*, *49*(6), 2204-2218.

Stadthagen-González, H., Ferré, P., Pérez-Sánchez, M. A., Imbault, C., & Hinojosa, J. A. (2018). Norms for 10,491 Spanish words for five discrete emotions: Happiness, disgust, anger, fear, and sadness. *Behavior Research Methods*, *50*(5), 1943–1952. doi: 10.3758/s13428-017-0962-y

Stadthagen-González, H., Imbault, C., Pérez-Sánchez, M. A., & Brysbaert, M. (2017). Norms of valence and arousal for 14,031 Spanish words. *Behavior Research Methods*, *49*(1), 111–123. doi: 10.3758/s13428-015-0700-2

Starostin, S. (2007). Opredelenije ustojčivosti bazisnoj leksiki [Determining the stability of basic words]. In *S. A. Starostin: Trudy po jazykoznaniju [S. A. Starostin: Works on linguistics* (p. 580-590). Moscow: Languages of Slavic Cultures.

Starostin, S. A. (2000). *The STARLING database program.* Moscow: RGGU. Retrieved from `http://starling.rinet.ru`

Swadesh, M. (1955). Towards greater accuracy in lexicostatistic dating. *International Journal of American Linguistics*, *21*(2), 121–137. doi: 10.1086/464321

Tadmor, U. (2009). Loanwords in the world's languages - Findings and results. In M. Haspelmath & U. Tadmor (Eds.), *Loanwords in the World's Languages* (pp. 55–75). Berlin, Germany: De Gruyter Mouton. doi: 10.1515/9783110218442.55

Thomas, D. (1960). Basic vocabulary in some Mon-Khmer languages. *Anthropological Linguistics*, *2*(3), 7-11.

Tjuka, A., Forkel, R., & List, J.-M. (2021). *NoRaRe. A database of cross-linguistic norms, ratings, and relations for words and concepts (Version 0.2).* Jena: Max Planck Institute for the Science of Human History. doi: 10.5281/zenodo.3957680

Tsang, Y.-K., Huang, J., Lui, M., Xue, M., Chan, Y.-W. F., Wang, S., & Chen, H.-C. (2018). MELD-SCH:

A megastudy of lexical decision in simplified Chinese. *Behavior Research Methods*, *50*(5), 1763–1777. doi: 10.3758/s13428-017-0944-0

van Heuven, W. J. B., Mandera, P., Keuleers, E., & Brysbaert, M. (2014). SUBTLEX-UK: A new and improved word frequency database for British English. *Quarterly Journal of Experimental Psychology*, *67*(6), 1176–1190. doi: 10.1080/17470218.2013.850521

Vejdemo, S., & Hörberg, T. (2016). Semantic factors predict the rate of lexical replacement of content words. *PLoS ONE*, *11*(1), 1–15. doi: 10.1371/journal.pone.0147924

Vergallito, A., Petilli, M. A., & Marelli, M. (2020). Perceptual modality norms for 1,121 Italian words: A comparison with concreteness and imageability scores and an analysis of their impact in word processing tasks. *Behavior Research Methods*, *52*(4), 1599–1616. doi: 10.3758/s13428-019-01337-8

Verheyen, S., De Deyne, S., Linsen, S., & Storms, G. (2020). Lexicosemantic, affective, and distributional norms for 1,000 Dutch adjectives. *Behavior Research Methods*, *52*, 1108–1121. doi: 10.3758/s13428-019-01303-4

Vulić, I., Baker, S., Ponti, E. M., Petti, U., Leviant, I., Wing, K., … Korhonen, A. (2020). Multi-SimLex: A large-scale evaluation of multilingual and cross-lingual lexical semantic similarity. *Computational Linguistics*, *46*(4), 1–51. doi: 10.1162/coli_a_00391

Warriner, A. B., Kuperman, V., & Brysbaert, M. (2013). Norms of valence, arousal, and dominance for 13,915 English lemmas. *Behavior Research Methods*, *45*(4), 1191–1207. doi: 10.3758/s13428-012-0314-x

Wierzba, M., Riegel, M., Wypych, M., Jednoróg, K., Turnau, P., Grabowska, A., & Marchewka, A. (2015). Basic emotions in the Nencki Affective Word List (NAWL BE): New method of classifying emotional stimuli. *PLoS ONE*, *10*(7), 1–16. doi: 10.1371/journal.pone.0132305

Wikimedia. (2020). *Wikidata, the free knowledge base.* Retrieved from `https://www.wikidata.org/`

Winter, B. (2016). Taste and smell words form an affectively loaded and emotionally flexible part of the English lexicon. *Language, Cognition and Neuroscience*, *31*(8), 975–988. doi: 10.1080/23273798.2016.1193619

Wu, W., Nicolai, G., & Yarowsky, D. (2020). Multilingual dictionary based construction of core vocabulary. In *Proceedings of the 12th Language Resources and Evaluation Conference* (pp. 4211–4217). Marseille, France: European Language Resources Association. Retrieved from `https://www.aclweb.org/anthology/2020.lrec-1.519`

Xiao, W., & Treiman, R. (2012). Iconicity of simple Chinese characters. *Behavior Research Methods*, *44*(4), 954–960. doi: 10.3758/s13428-012-0191-3

Yao, Z., Wu, J., Zhang, Y., & Wang, Z. (2017). Norms of valence, arousal, concreteness, familiarity, imageability, and context availability for 1,100 Chinese words. *Behavior Research Methods*, *49*(4), 1374–1385. doi: 10.3758/s13428-016-0793-2

Zalizniak, A. A., Smirnitskaya, A., Russo, M., Mikhailova, T., Bobrik, M., Gruntov, I., … Voronov, M. (2020). *Database of semantic shifts (version from 07/10/2020).* Moscow: Institute of Linguistics at the Russian Academy of Sciences. Retrieved from `http://datsemshift.ru/`
